# A rare case of dermatomyositis revealed during pregnancy with good outcome

**DOI:** 10.11604/pamj.2016.23.117.9198

**Published:** 2016-03-24

**Authors:** Kelati Awatef, Gallouj Salim, Mernissi Fatima Zahra

**Affiliations:** 1 University Hospital Hassan II of Fez, Department of Dermatology, Morocco

**Keywords:** Dermatomyositis, pregnancy, good outcome

## Abstract

There are only few case reports in the literature documenting outcome of pregnancy in patients with DM in contrast with those of other connective tissue diseases especially when dermatomyositis is revealed during pregnancy, most of the publications reported a poor prognosis for both the mother and the fetus, yet, in our case we confirmed the results of the rare recent cases that have tended to show a good outcome, after the treatment with glucocorticoid and immunosuppressant therapy.

## Introduction

Dermatomyositis (DM) is an idiopathic connective tissue disease characterized by specific cutaneous findings and inflammatory lesions in the muscle biopsy [1]. The onset of dermatomyositis during pregnancy is a rare event [2]. 17% of DM known before pregnancy will relapse during the pregnancy period and when the DM begins or relapses during pregnancy, the prognosis is pejorative with fetal death in 50% of cases, but successful therapy will permit a satisfactory result [3]. We report a rare case of DM revealed during pregnancy with a good outcome after using oral corticosteroids.

## Patient and observation

**Figure 1:**
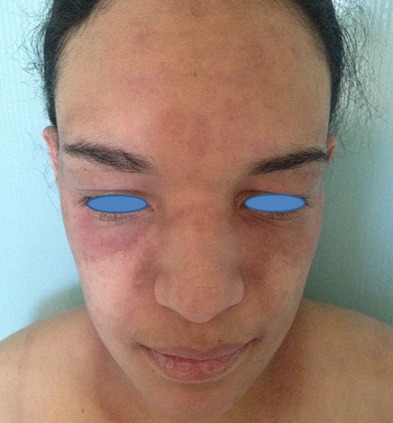
DM showing pink liliaceperiocularerythro edema of the face

**Figure 2:**
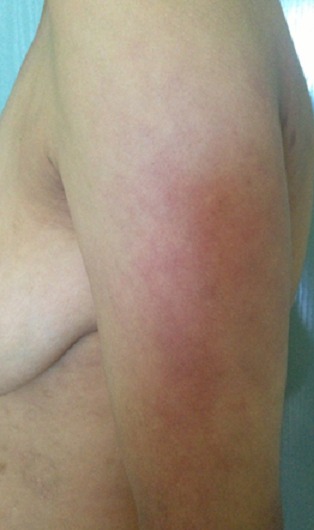
DM showing purplish erythema of the buttocks

**Figure 3:**
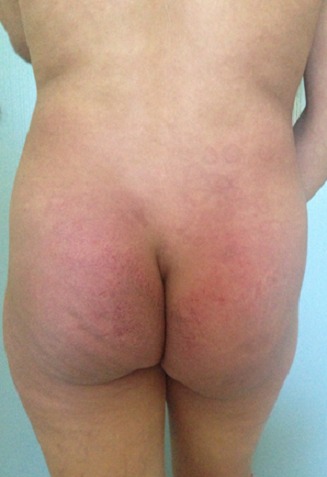
DM showing erythema of the external face of the thighs

**Figure 4:**
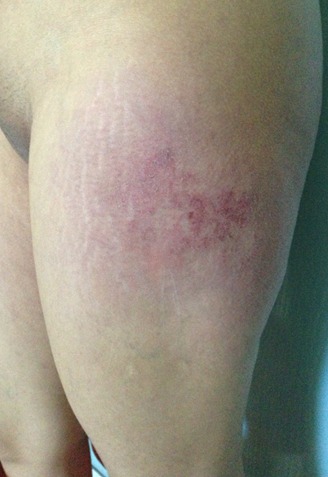
DM showing erythema of the external face of the arms

**Figure 5:**
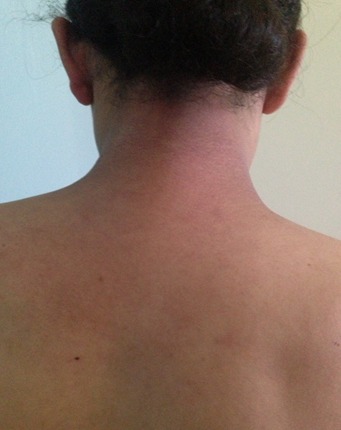
DM showing purplish erythema of the neck and neckline

A 28-year-old woman in the third month of pregnancy, who presented with a pruritic rash of the face, hands, buttocks and the thighs that appeared at 4 weeks of gestation. She had not taken any medicine before the rash appeared. She did not complain of muscle pain, with a slight fatigue and there was no personal or family history of connective tissue disease. Examination revealed a pink liliace periocular erythro edema of the face (Figure 1), pruritic papules on the dorsal hands, elbows and a purplish erythema of the buttocks (Figure 2), the external surface of the thighs (Figure 3) and arms (Figure 4), neck and neckline (Figure 5), congestive erythema of the posterior nail fold with Periungual telangiectasia and hyperkeratosis of the cuticle were also observed, neuromuscular examination revealed a slight deficit of the pelvic girdle. Results of complete blood counts, blood biochemistry analysis, and urine analysis were within normal limits. Although antinuclear antibody titer (1:160) was positive, other auto antibodies were negative. A skin and muscle biopsy was compatible with DM with electric signs confirming the DM in the electromyogram. Based on these findings, this patient was diagnosed as DM revealed during pregnancy. Potent topical steroids with Hydroxychloroquine were prescribed, but the eruption did not improve with exacerbation of the erythroedema of the skin and the muscle weakness and the elevation of muscle enzymes, so we prescribed corticosteroid therapy for the patient with good evolution and she delivered a healthy newborn at term. The rash began to disappear progressively after delivery, so, the dose reduction of the corticosteroids was maintained gradually; also; both the mother and the newborn were plugged into the consultation department for regular monitoring.

## Discussion

Dermatomyositis is a rare medical disease complicating pregnancy [4], various factors have been considered as triggers for development of DM during pregnancy; for example, exposure of the mother to fetal antigens, changes in maternal hormonal status, and the reactivation of certain viruses by pregnancy. Recently it has been proposed that microchimerism may contribute to the pathogenesis of autoimmune diseases [5–8]. There are only few case reports in the literature documenting outcome of pregnancy in patients with DM in contrast with those of other connective tissue diseases, yet, most of the publications reported a poor prognosis for both the mother and the fetus; maternal death has been described and attached to complications of hypertension and the disease evolution [9]. In addition, prematurity may occur or even fetal death in 50% of cases [10–13] or the occurrence of Juvenile DM after post partum [14]. Some authors suggest that the outlook for the fetus is unfavorable when DM is first diagnosed during pregnancy [15], for example, in the study of Gutiearrez et al [16] of 18 women with polymyositis/ dermatomyositis, there was 55% of increase in the rate of fetal loss and 50% of pregnancies ended prematurely and there was no correlation between disease activity and fetal loss.

Other authors consider that fetal prognosis parallels the activity of maternal disease, which was also reported in the study of Silva et al [17] who published a more detailed series of pregnancy in 28 women with dermatomyositis and polymyositis and concluded that the more active the myositis during pregnancy, the greater the chances of fetal loss. Generally, Optimal pregnancy success can be anticipated when pregnancy is undertaken with disease in remission. Some rare cases [18, 19] have tended to show a good outcome, after the treatment with oral corticosteroids. The use of steroids as first-line therapy is indicated for maternal disease exacerbation during pregnancy, as in the non-pregnant state. Other chemotherapeutic agents, such as intravenous immunoglobulins and antineoplastic agents can be used as second-line drugs to control disease activity and to maintain pregnancy.

These cases and studies concerns mostly patients followed for DM and became pregnant, but there is only a few data concerning the management, the fetal and maternal prognosis in DM revealed during pregnancy. For example, in the largest series reported in the literature, there were only 2 patients among 98 who developed dermatomyositis during pregnancy with good control of the disease after glucocorticoid and immunosuppressant therapy [20]. In our case, we administered corticosteroids after the failure of steroid ointment and Hydroxychloroquine with good outcome, and the prospective maternal and fetal evaluation is undertaken.

## Conclusion

Our case documents the theory that, DM onset during pregnancy had good outcome after drug therapy especially corticosteroids.

## References

[1] Papakonstantinou E, Kapp A, Raap U (2016). A mildform of dermatomyositis as a prodromalsign of lungadenocarcinoma: a case report. J Med Case Rep..

[2] Méaux-Ruault N, Gil H, Curlier E, Magy-Bertrand N (2010). Dermatomyosite révélée au cours d'une grossesse et guérie en post-partum. La Revue de médecine interne.

[3] Chopra S, Suri V, Bagga R, Thami MR, Sharma A, Bambery P (2008). Autoimmune inflammatory myopathy in pregnancy. Medscape J Med.

[4] King CR, Chow S (1985). Dermatomyositis and pregnancy. Obstet Gynecol..

[5] Morihara K, Katoh N (2004). Amyopathic dermatomyositis presenting during pregnancy. JAAD.

[6] Katz AL (1980). Another case of polymyositis in pregnancy. Arch Intern Med.

[7] Tsai A, Lindheimer MD, Lamberg SI (1973). Dermatomyositis completing pregnancy. Obstet Gynecol.

[8] Satoh M, Ajmani AK, Hirakata M, Suwa A, Winfield JB, Reeves WH (1994). Onset of polymyositis with autoantibodies to threonyl tRNA synthetase during pregnancy. J Rheumatol.

[9] Huong DLT, Wechsler B (2005). Maladies systémiques pendant la grossesse. Revue du Rhumatisme..

[10] Gutierrez G, Dagnino R, Mintz G (1984). Polymyositis/dermatomyositis and pregnancy. Arthritis Rheum.

[11] Kofteridis DP, Malliotakis PI, Stsiu F, Vardakis NK, Vamvakas LN, Emmanouel DS (1999). Acute onset of dermatomyositis presenting in pregnancy with rhabdomyolysis and fetal loss. Scand J Rheumatol..

[12] Satoh M, Ajmani AK, Hirakata M, Suwa A, Winfield JB, Reeves WH (1994). Onset of polymyositis with autoantibodies to threonyl-tRNAsynthetase during pregnancy. J Rheumatol.

[13] Harris A, Webley M, Usherwood M, Burge D (1995). Dermatomyositispresenting in pregnancy. Br J Dermatol.

[14] Madu AE, Omih E, Baguley E, Lindow SW (2013). Juvenile Dermatomyositis in Pregnancy. Case Rep Obstet Gynecol.

[15] Rosenzweig BA, Rotmensch S, Binette SP, Phillippe M (1989). Primary idiopathic polymyositis and dermatomyositis complicating pregnancy: diagnosis and management. Obstet Gynecol Surv.

[16] Gutiearrez G, Dagnino R, Mintz G (1984). “Polymyositis/dermatomyositis andpregnancy”. Arthritis&Rheumatism..

[17] Silva CA, Sultan SM, Isenberg DA (2003). Pregnancy outcome in adult-onset idiopathic inflammatory myopathy. Rhematology.

[18] Ohno T, Imai A, Tamaya T (1992). Successful outcomes of pregnancy complicated with dermatomyositis: case reports. Gynecol Obstet Invest..

[19] Kaddour N, Snoussi M (2012). Pregnancy in dermatomyositis and polymyositis. Tunis Med.

[20] Missumi LS, Souza FH, Andrade JQ, Shinjo SK (2015). Pregnancy outcomes in dermatomyositis and polymyositis patients. Rev Bras Reumatol..

